# Spatially distinct molecular patterns of gene expression in idiopathic pulmonary fibrosis

**DOI:** 10.1186/s12931-023-02572-6

**Published:** 2023-11-17

**Authors:** Rachel Z. Blumhagen, Jonathan S. Kurche, Carlyne D. Cool, Avram D. Walts, David Heinz, Tasha E. Fingerlin, Ivana V. Yang, David A. Schwartz

**Affiliations:** 1 Center for Genes, Environment and Health, National Jewish Health, 1400 Jackson St, Office M222D, Denver, CO 80206 USA; 2 Department of Medicine, University of Colorado Anschutz Medical Campus, 13001 E. 17th Place, Aurora, CO 80045 USA; 3 Medical Service, Rocky Mountain Regional Veterans Administration Medical Center, 1700 N Wheeling St, Aurora, CO 80045 USA; 4 Department of Medicine, National Jewish Health, 1400 Jackson St, Denver, CO 80206 USA; 5 Pathology Laboratory, National Jewish Health, 1400 Jackson St., Denver, CO 80206 USA

## Abstract

**Background:**

Idiopathic pulmonary fibrosis (IPF) is a heterogeneous disease that is pathologically characterized by areas of normal-appearing lung parenchyma, active fibrosis (transition zones including fibroblastic foci) and dense fibrosis. Defining transcriptional differences between these pathologically heterogeneous regions of the IPF lung is critical to understanding the distribution and extent of fibrotic lung disease and identifying potential therapeutic targets. Application of a spatial transcriptomics platform would provide more detailed spatial resolution of transcriptional signals compared to previous single cell or bulk RNA-Seq studies.

**Methods:**

We performed spatial transcriptomics using GeoMx Nanostring Digital Spatial Profiling on formalin-fixed paraffin-embedded (FFPE) tissue from 32 IPF and 12 control subjects and identified 231 regions of interest (ROIs). We compared normal-appearing lung parenchyma and airways between IPF and controls with histologically normal lung tissue, as well as histologically distinct regions within IPF (normal-appearing lung parenchyma, transition zones containing fibroblastic foci, areas of dense fibrosis, and honeycomb epithelium metaplasia).

**Results:**

We identified 254 differentially expressed genes (DEGs) between IPF and controls in histologically normal-appearing regions of lung parenchyma; pathway analysis identified disease processes such as EIF2 signaling (important for cap-dependent mRNA translation), epithelial adherens junction signaling, HIF1α signaling, and integrin signaling. Within IPF, we identified 173 DEGs between transition and normal-appearing lung parenchyma and 198 DEGs between dense fibrosis and normal lung parenchyma; pathways dysregulated in both transition and dense fibrotic areas include EIF2 signaling pathway activation (upstream of endoplasmic reticulum (ER) stress proteins ATF4 and CHOP) and wound healing signaling pathway deactivation. Through cell deconvolution of transcriptome data and immunofluorescence staining, we confirmed loss of alveolar parenchymal signals (AGER, SFTPB, SFTPC), gain of secretory cell markers (SCGB3A2, MUC5B) as well as dysregulation of the upstream regulator ATF4, in histologically normal-appearing tissue in IPF.

**Conclusions:**

Our findings demonstrate that histologically normal-appearing regions from the IPF lung are transcriptionally distinct when compared to similar lung tissue from controls with histologically normal lung tissue, and that transition zones and areas of dense fibrosis within the IPF lung demonstrate activation of ER stress and deactivation of wound healing pathways.

**Supplementary Information:**

The online version contains supplementary material available at 10.1186/s12931-023-02572-6.

## Introduction

Idiopathic pulmonary fibrosis (IPF) is a progressive and fatal disease of the aging lung [1, 2] that is increasing in prevalence [3] and is likely underdiagnosed [4, 5]. IPF is heterogeneous pathologically with patchy areas of fibrosis mixed with areas of normal lung tissue [2, 6]. Pathologically, IPF is characterized by the usual interstitial pneumonia (UIP) pattern that includes normal appearing lung tissue adjacent to active areas of fibroproliferation (fibroblastic foci) and microscopic honeycomb lesions [7]. Although areas of IPF lung parenchyma without evidence of lung fibrosis have recently been shown to have a drop-out of small airways [8], the biological activity of normal appearing regions of IPF lung are only beginning to be studied [9].

Previous investigations of transcriptional profiles of IPF lung have largely been performed on whole lung tissue and have identified several thousand genes that are differentially regulated in IPF [10–17]; consistently reported genes and pathways include extracellular matrix organization and regulation, TGF-β signaling, endoplasmic reticulum stress, epithelial-mesenchymal transition (EMT), mitochondrial homeostasis, bronchiolar epithelial genes, fibroblast genes, smooth muscle markers, cytokines and chemokines, growth factors and receptors. More recent single cell studies [18–21] identified novel cell populations that appear to have a functional role in disease pathogenesis, such as *KRT17*-expressing “aberrant basaloid” cells [18] and “secretory-primed basal (SPB)” [20] cells. However, a study combining microCT assessment of the extent of fibrosis and bulk transcriptomic analysis identified unique transcriptional profiles associated with the extent of fibrosis [22], suggesting that regional analysis of heterogeneous lung tissue may provide novel insights in this progressive disease.

**Fig. 1:**
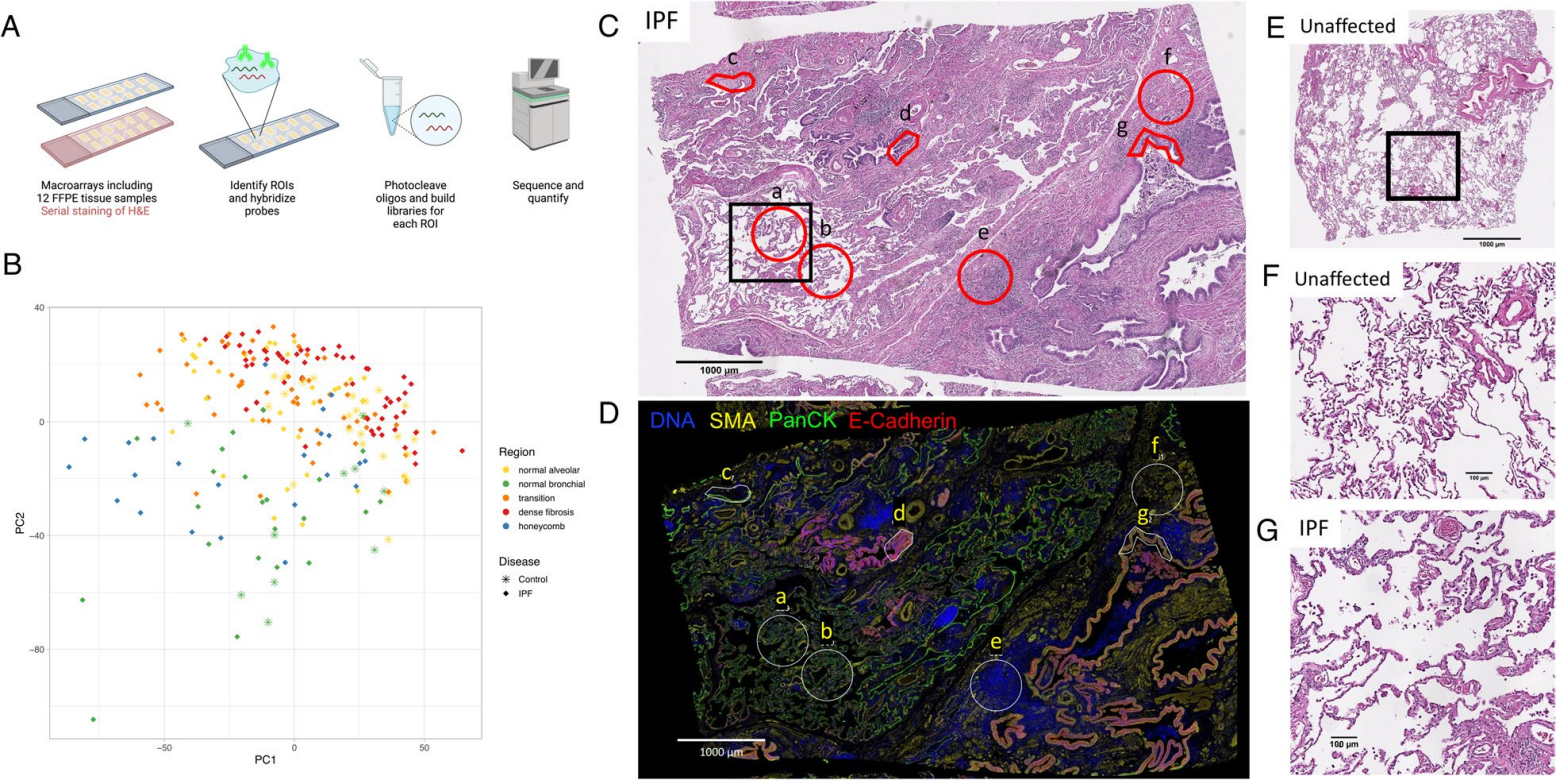
Overview of the GeoMx platform (**A**). PCA plot by disease and region (**B**). Example ROI selection of tissue from an IPF subject, H&E (**C**) with corresponding immunofluorescence (**D**) showing regions of normal appearing lung parenchyma (a,b), normal bronchiolar epithelium (c), transition (d), dense fibrosis (e,f) and honeycomb epithelium (g). H&E showing regions of normal appearing lung parenchyma at higher magnification in a control with histologically normal lung tissue (**E**, **F**) and IPF (**G**)

In the past several years, spatial transcriptomics technology has emerged as an approach to capture whole transcriptome information from histological or morphological regions of interest. These methods can be applied to frozen or FFPE tissue allowing for application to a broad range of studies involving patient samples. The general approach is to quantify RNA in FFPE tissue using probe-based mount specimens in which slides are stained directly with relevant morphology markers or serial staining with H&E. The slides are then imaged for selection of regions of interest (ROI) via a software platform (Fig. 1). The platform allows capturing ROI using various approaches (grid selection, circular or geometric region selection) to allow for highly specific capture of histological features of interest. The resulting data provide whole transcriptome quantification for each ROI that can be compared across region types or other biological factors.

A recent application of spatial transcriptomic technology in IPF focused on alveolar regions, fibroblastic foci, immune infiltrates, and blood vessel regions, however, this study was limited to only 10 cases of IPF [23]. In addition to showing distinct gene expression signatures and cell population variation between regions of interest, the authors identified reduced inflammatory gene signatures in the fibrotic niche, and reduced Type I interferon response in alveolar septae when comparing IPF to controls with histologically normal lung tissue. Here we report a more comprehensive study of IPF lung tissue utilizing spatial transcriptomic technology to compare normal appearing lung parenchyma and airways between IPF and controls with histologically normal lung tissue, as well as an analysis of histologically distinct regions within the IPF lung.

## Methods

### Overview

We used the Nanostring GeoMX Digital Spatial Profiler to obtain spatial transcriptomic data on histologically different regions of interest (ROIs) from FFPE tissue from IPF subjects and controls with histologically normal lung tissue. We compared whole transcriptome expression between IPF subjects and controls with histologically normal lung tissue within histologically normal tissue types (normal lung parenchyma and normal bronchiolar epithelium). In addition, we compared increasingly diseased tissue regions to their comparative normal tissue in IPF subjects; dense fibrosis and transition zones which included fibroblastic foci were compared to normal appearing lung parenchyma, and honeycomb epithelial metaplasia was compared to normal appearing bronchiolar epithelium. To determine transcriptional differences independent of differences in cell composition, we estimated cell proportions of human lung cell types relevant to IPF by applying the cell deconvolution method, SpatialDecon [24]. We performed differential expression adjusting for a subset of cell types identified as having the largest variation and influence on comparisons of interest. To identify pathways, networks, and upstream regulators enriched among the differentially expressed genes, we used Ingenuity Pathway Analysis (IPA) and Network Analyst on the differential expression results [25, 26]. For validation of the spatial transcriptomics data, we performed immunofluorescence microscopy using the same macroarrays included in the GeoMx assays and compared normal parenchyma regions between control and IPF subjects.

### Lung tissue samples and sample preparation

Lung tissue was obtained from whole explants or surgical lung biopsies from 32 subjects with IPF and 12 controls with histologically normal lung tissue. De-identified data and samples were approved for use in this study by the Colorado Multiple Institutional Review Board (COMIRB #15-1147). Patients were consented and tissue was collected through the NHLBI-sponsored Lung Tissue Research Consortium (LTRC) and at the University of Colorado. IPF was diagnosed in the LTRC using American Thoracic Society/European respiratory Society (ATS/ERS) criteria [7] and final LTRC diagnosis was based on an integrated clinical, radiologic, and pathologic diagnosis. Samples collected at the University of Colorado followed similar protocols to the LTRC for diagnosis and collection of clinical data and lung tissue. Each sample was reviewed by our lung pathologist (C.D.C). Control tissue was histologically normal tissue isolated with no evidence of metaplasia or lung cancer features identified from lung cancer resections through the LTRC and hereafter are referred to as ‘controls with histologically normal lung tissue’. Tissue from patients with IPF fulfilled the pathological criteria of UIP. Each slide was further subselected (5 × 5 mm^2^) for areas of classic UIP appearance or normal lung in controls with histologically normal lung tissue. The corresponding areas in the formalin-fixed paraffin-embedded (FFPE) tissue blocks were dissected and a new block consisting of up to 12 subjects was prepared. These macroarray blocks (Additional file 1: Fig. S1) were then cut at 4 μm thickness and stained with H & E.

### Nanostring geoMX digital spatial profiler

We used the Nanostring GeoMX Digital Spatial Profiler (DSP) platform to obtain human whole transcriptome data from lung tissue macroarrays [27]. Region of interest (ROI) selection was performed on immunoflourescently stained tissue for morphology markers pan-cytokeratin, E-cadherin, α-smooth muscle actin (SMA), and DNA. Importantly, serially cut H&E slides were used by our pathologist to guide ROI selection. Normal parenchymal tissue was characterized by relatively thin and delicate alveoli (Fig. 1C, E–G). Transition-type tissue demonstrated areas of septal thickening and fibroblastic foci adjacent to near-normal alveolar epithelium. The fibroblastic areas were highlighted by the SMA immunofluorescent stain. Densely fibrotic areas consisted of collagen admixed with variable degrees of chronic inflammation. The densely fibrotic areas were generally less cellular, which was confirmed by fewer nuclei visible by DNA immunofluorescence. Bronchiolar epithelium was determined by the presence of ciliated columnar epithelium surrounded by circumferential smooth muscle on H & E and confirmed with positive immunofluorescent staining for pancytokeratin. Honeycomb metaplastic epithelium consisted of dilated spaces lined by squamous-to-columnar epithelium, often filled with mucus, and surrounded by disorganized, non-circumferential fibrosis. The epithelium of the honeycomb cysts was highlighted by the pan-cytokeratin marker. ROIs featuring normal lung parenchyma, transition, or densely fibrotic areas were drawn using the ellipse drawing tool while ROIs capturing normal bronchiolar and honeycomb metaplasia epithelium were drawn using the freeform drawing tool to focus on the bronchiolar-like epithelium. Examples of selected ROIs are shown (Fig. 1D).

### QC and normalization

We followed the GeoMx RNA-Seq pipeline to convert raw DCC files to normalized expression data [28–30]. We kept samples having greater than 1000 reads, 80% reads trimmed, 80% reads stitched, 70% reads aligned, and sequencing saturation above 50%. To remove genes with low signal to noise ratio, we retained targets with expression above the limit of quantification (LOQ) score (defined in (1)) in 2% or more of the total number of samples that were included. We performed Q3 normalization based on all remaining gene targets. All analyses were performed using R version 4.1.2 (2021-11-01) [31].

1 $$LOQ_{i} = geomean\,(NegProbe_{i} )\,*\,geoSD\,(NegProbe_{i} )^{2}$$

### Statistical analysis

Principal component analysis was performed on the log2 Q3 normalized counts. To perform differential expression, we modeled the log2 Q3 normalized counts using linear mixed effects model with a categorical variable representing disease and region as the main predictor (Eq. 2). Based on initial QC and investigation of PCA plots, all models were adjusted for batch based on day of macrorray processing. We included a random effect for subject to account for the correlation of ROIs within the same subject.

$${Gene}_{ij}= {\beta }_{0}+{\beta }_{Group}\times {Group}_{ij }+ {\alpha }_{i}+ {\epsilon }_{ij}$$

2 $$\varepsilon _{{ij~}} \sim ~N\left( {0,\sigma ^{2} } \right),\alpha _{i} \sim ~N\left( {0,\sigma _{{a_{j} }}^{2} } \right),~\quad {\text{i}} = 1,2, \ldots ,{\text{m}};{\text{j}} = 1,2, \ldots ,{\text{M}}$$

Gene _*ij*_ is the Q3 normalized expression data for a given gene for sample *i* for subject *j*. *β*_*Group*_ represents the coefficient for the fixed effect term *Group*_*ij*_. *Group*_*ij*_ is the disease region variable which included control normal lung parenchyma, control normal bronchiolar epithelium, IPF normal lung parenchyma, IPF normal bronchiolar epithelium, IPF transition, IPF dense fibrosis and IPF honeycomb epithelium metaplasia. We applied contrast statements to compute differential expression between groups of interest. For the disease-focused research question, we compared IPF to control within normal lung parenchyma and normal bronchiolar epithelium. For the region focused research question within IPF, we compared normal lung parenchyma tissue to transition and dense fibrosis tissue and compared normal bronchiolar epithelium to honeycomb epithelium. To adjust for multiple comparisons, we applied a Benjamini-Hochberg adjustment where genes with an FDR adjusted p-value < 0.05 were considered significant. We did not consider genes significant if the model was determined to be singular. Linear mixed models were computed using the *lmerSeq* R package [32].

### Cell deconvolution analysis

To examine how the selected ROIs differ in the composition of different lung cell types, we applied a cell deconvolution method, SpatialDecon, developed specifically for spatial transcriptomic data [24]. This method uses a log mean deconvolution algorithm which is more appropriate for the distribution of counts characteristic of GeoMx data. We deconvolved the spatial data using two references: (1) a normal human lung cell atlas which included marker gene lists for 21 cell types [33] and (2) an IPF lung cell atlas of 38 cell types [18]. We applied the functions from the SpatialDecon R package to the Q3 normalized counts and the background correction factors computed from the negative probes. To examine differences in the proportions of IPF cell types, we modeled log-transformed proportions by disease regions adjusting for batch as a covariate and subject as a random effect using a linear mixed effects model. In the same way we tested expression, we tested for changes in proportions between groups for our comparisons of interest using contrast statements; p-values were adjusted for multiple comparisons across cell types with a Benjamini-Hochberg adjustment. We removed cell types estimated as 5% or less in more than 95% of samples. Prior to log transformation, proportions equal to zero were set at 1/1000.

To adjust for differences in cell composition, we applied the linear mixed model outlined above and included the estimated cell proportions as covariates. Our primary differential expression analysis adjusted for cell types estimated from the normal human lung reference and selected based on biological relevance in IPF pathology (alveolar epithelial cells Type 1(AEC1), alveolar epithelial cells Type 2 (AEC2), fibroblasts and ciliated cells).

### Pathway analysis

We applied the Ingenuity Pathways Analysis [25, 26] to the differential expression results adjusted for batch and cell proportions of AEC1, AEC2, fibroblasts, and ciliated cells based on the normal human lung reference.

### Immunofluorescence and quantification

For validation of GeoMx data, immunofluorescence microscopy was used to compare normal alveolar regions from control and IPF tissue using the same macroarrays included in the GeoMx assays above. Samples were deparaffinized using xylene and antigens retrieved by boiling in 10mM citrate, pH 6.0. Specimens were blocked for 1 h using 2.5% BSA in PBS, then labelled with specific antibodies overnight in PBS containing 0.25% BSA at 4̊C. Slides were washed 3x with 0.1% Triton-X100 in PBS, then labelled with secondary antibodies for one hour at room temperature. Samples were then washed 3x again in 0.1% Triton-X100 in PBS, washed again to remove detergent, and stained with DAPI, then mounted with Floromount G (Southern Biotech). For a complete list of antibodies and concentrations used in these studies, see Additional file 1:  Table S2. For each sample, nine adjacent 20x immunofluorescence images arranged in a 3 × 3 grid were acquired based on typical alveolar features selected at random using a Keyence BZ-X800 inverted microscope. Composite images were stitched together using the manufacturer’s BZ-H4A software and exported as TIFs for further processing in ImageJ. Fluorescent labels were thresholded to eliminate background, converted to masks, and quantified by area and number of fluorescent foci. Measurements were normalized according to the numbers of nuclei present in the image. Data were exported to PRISM where differences in normalized area between IPF and controls with histologically normal lung tissue were tested according to Mann-Whitney U test and statistical significance was set at p < 0.05.

## Results

We selected a total of 231 ROIs from 32 IPF subjects (ROI N = 196) and 12 controls with histologically normal lung tissue (ROI N = 35). The distribution of age, sex, smoking status and race were comparable between IPF subjects and controls with histologically normal lung tissue (Table 1). ROIs from controls with histologically normal lung tissue consisted of normal tissue types (normal parenchyma and normal bronchiolar epithelium) and ROIs from IPF subjects consisted of normal appearing parenchyma and bronchiolar epithelium in addition to disease tissue (transition, dense fibrosis, and honeycomb epithelium metaplasia) (Table 1). We analyzed 10,262 genes after removing 8415 genes that had a low signal to noise ratio. We observed variation in expression captured by disease status and tissue type as evidenced by principal component analysis separating samples by disease (PC1) and region type (PC2) (Fig. 1). Additionally, we identified separation of PCs by batch (indicator for the day the tissue macroarrays were processed), and consequently adjusted for batch in all models (Additional file 1:  Fig. S2).

**Table 1:** Summary of subject demographics information

Demographics	Controls with histologically normal lung tissue (N = 12)	IPF (N = 32)
Sex		
Male	7 (58)	22 (69)
Female	5 (42)	10 (31)
Age		
Min	28	36
Median	64.5	65
Max	74	78
Mean (SD)	60.00 (12.88)	61.91 (8.89)
Smoker		
Yes	9 (75)	21 (66)
No	3 (25)	11 (34)
Race		
White	11 (92)	28 (88)
Black	0 (0)	3 (9)
Asian	1 (8)	0 (0)
Unknown	0 (0)	1 (3)
Ethnicity		
Non-Hispanic	12 (100)	30 (94)
Hispanic	0 (0)	1 (3)
Unknown	0 (0)	1 (3)
Comorbidities		
Angina	1 (8)	5 (16)
Heart failure	0 (0)	1 (3)
Arrhythmia	2 (17)	5 (16)
Hyperlipidemia	3 (25)	13 (41)
Diabetes	3 (25)	6 (19)
Lung cancer	2 (17)	0 (0)
Other cancer	4 (33)	2 (6)
Rheumatoid arthritis	0 (0)	2 (6)
Gerd	4 (33)	11 (34)
Asthma	0 (0)	1 (3)
Pulmonary hypertension	1 (8)	0 (0)
Emphysema	2 (17)	3 (9)
rs35705950 genotype		
GG	5 (42)	13 (41)
GT	6 (50)	16 (50)
TT	1 (8)	3 (9)
Summary of ROIs	# regions (# subj)	# regions (# subj)
Normal lung parenchyma	25 (12)	33 (27)
Normal bronchiolar epithelium	10 (10)	22 (22)
Transition		59 (32)
Dense fibrosis		58 (32)
Honeycomb metaplasia		24 (19)

### Cell deconvolution identifies differences in cell proportions

**Fig. 2:**
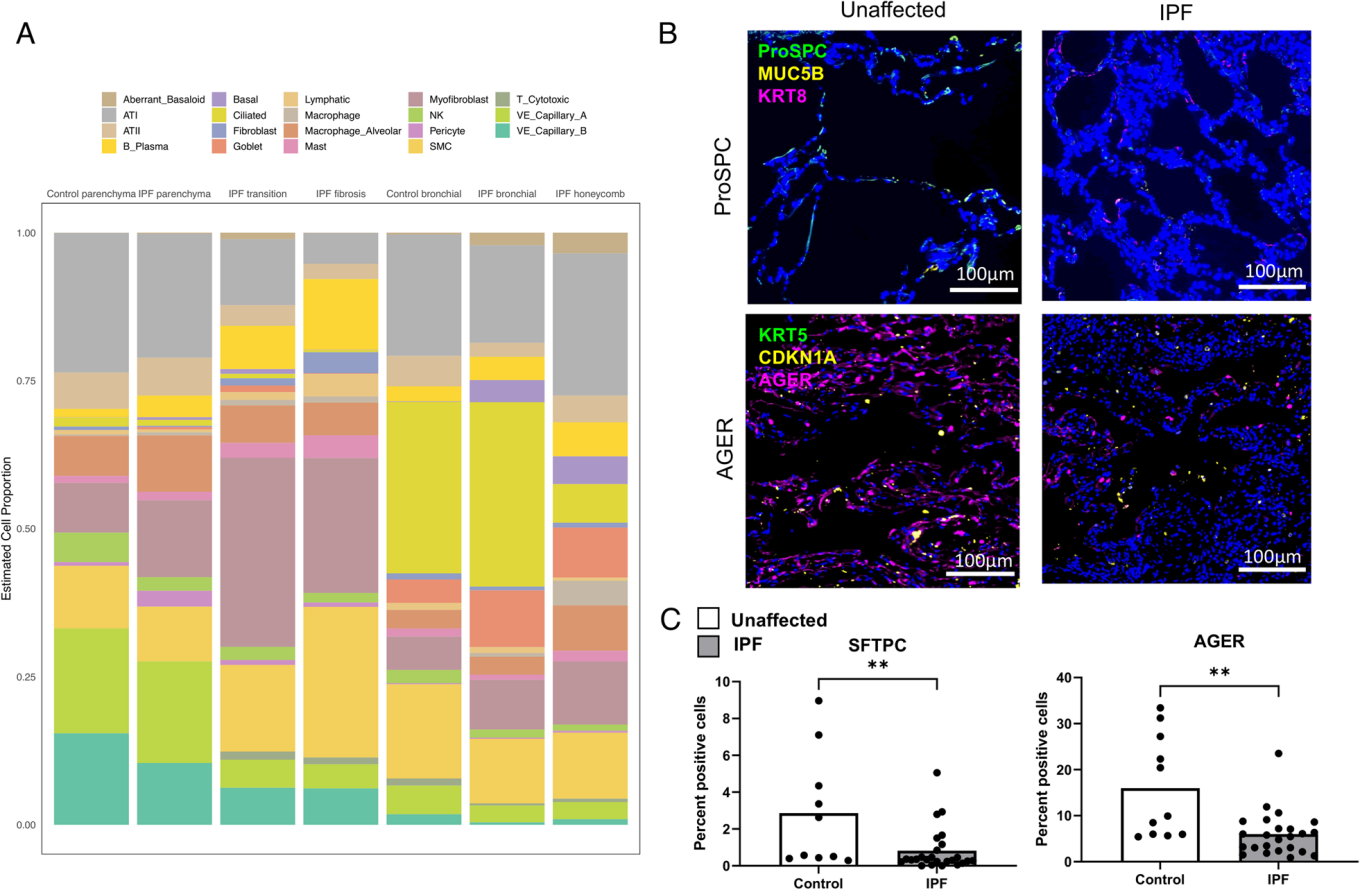
Mean cell proportions estimated with SpatialDecon based on IPF cell reference (**A**). Histology image showing changes in alveolar cell composition (**B**). Immunofluorescence changes in SFTPC and AGER between IPF and controls with histologically normal lung tissue in normal parenchyma regions (**C**). FOVs were chosen based on presence of features of interest (e.g. parenchyma, bronchiolar, etc.) in each section and 9 20x images centered randomly within the feature of interest were assembled into a composite. Pairwise comparisons are Mann-Whitney U test with significance set to (*) p < 0.05, (**) p < 0.01, (***) p < 0.001, (****) p < 0.0001

We examined estimated cell proportions of 10 cell types from the normal human lung reference and 19 cell types from the IPF diseased reference panel (Additional file 1:  Fig. S3). Among the 19 IPF cell types that were observed with the IPF reference, we observed fewer ciliated cells (p = 0.007) and greater pericytes (p = 7 × 10^−5^) between IPF and controls with histologically normal lung tissue in the normal parenchymal regions (Fig. 2A, Additional file 1: Table S2). There were no significant differences in cell proportions between IPF and controls with histologically normal lung tissue in the normal bronchiolar regions. In contrast, we observed greater changes in cell composition within IPF samples between histologically different regions. We observed an increased proportion of B plasma cells, fibroblasts, myofibroblasts, lymphatic cells, mast cells, smooth muscle cells and cytotoxic T cells in transition and dense fibrosis regions compared to normal parenchyma. There was a significant decrease in the proportion of alveolar epithelial cells (AEC1 and AEC2), vascular cells (VE Capillary A and B cells) and pericytes. Additionally, we found a significant increase in aberrant basaloid cells from normal parenchyma to transition but not for the comparison with regions of dense fibrosis.

The cellular composition based on the normal human lung reference were comparable to that of the IPF diseased reference panel, in which the composition of normal parenchymal and normal bronchiolar regions between IPF and control subjects were similar (Additional file 1: Fig. S3). As expected, we observed changes in cell composition across the histologically different regions; AEC1 and AEC2 were highest in the normal and non-fibrotic samples (control and IPF) and lower in disease regions within IPF subjects, and fibroblasts were observed at the highest proportions in the transition and dense fibrosis samples. We also observed a substantial increase in the proportion of ciliated cells in normal bronchiolar epithelium and honeycomb epithelium metaplasia.

We leveraged immunofluorescence microscopy to validate cell type predictions derived from spatial deconvolution. Using the same tissue macroarrays we observed reductions in AEC2 markers SFTPC and STFPB, as well as the AECI marker AGER (Fig. 2B and C; Additional file 1: Fig. S6). Based on these results, we chose to adjust for the estimated cell proportion of these four potentially influential cell types (AEC1, AEC2, fibroblasts, and ciliated cells).

*Transcriptional changes observed between IPF and controls with histologically normal lung tissue in non-fibrotic lung tissue*.

**Fig. 3:**
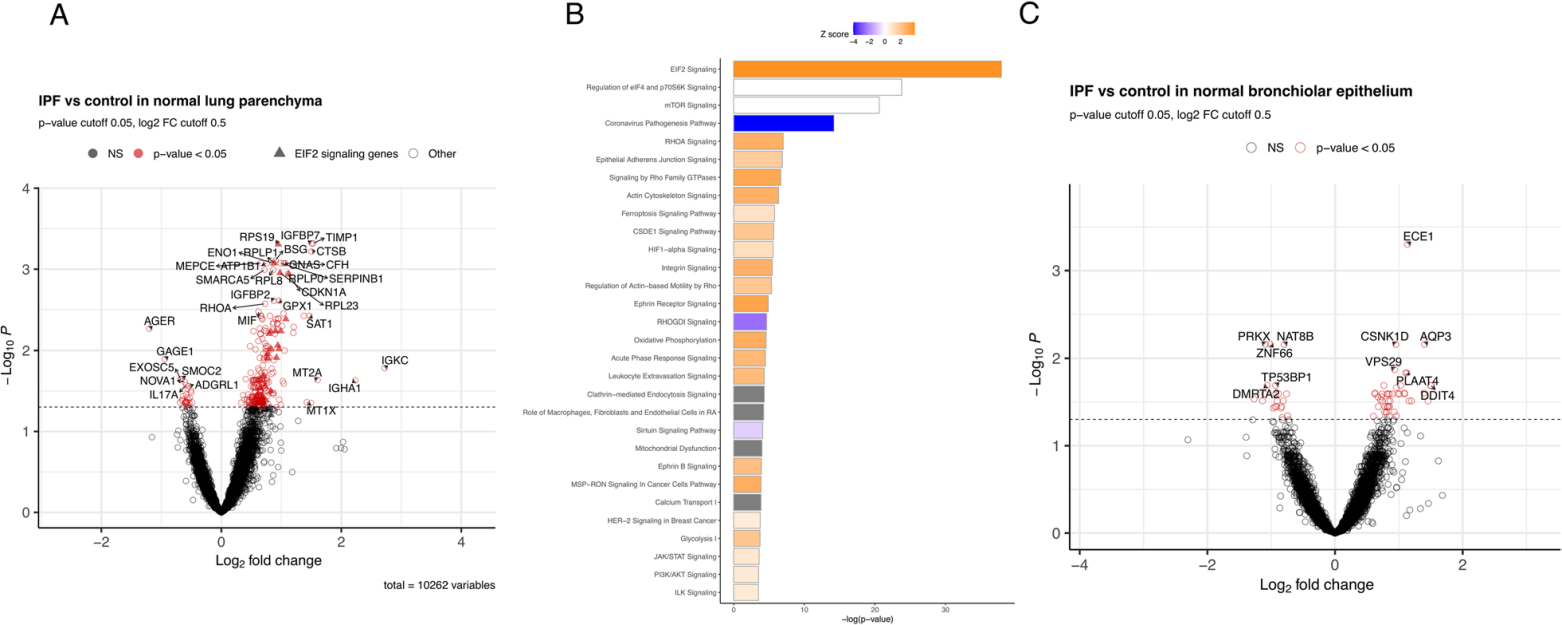
Differential expression between IPF and controls with histologically normal lung tissue in normal appearing lung parenchyma (**A**). Top 15 Ingenuity canonical pathways in the IPF vs. control normal lung parenchyma comparison (**B**). The activation z-score is a statistical measure based on the directional relationships between genes and their biological function. Orange indicates increased predictions over that of the null (positive z score), blue indicates decreased predictions (negative z score) and white z score of zero. Differential expression between IPF and controls with histologically normal lung tissue in normal bronchiolar regions (**C**) adjusting for batch and estimated cell proportions of AEC1, AEC2, fibroblasts and ciliated cells

To understand the transcriptional changes in non-fibrotic lung tissue in individuals with IPF compared to controls with histologically normal lung tissue, we compared expression in histologically normal appearing regions between IPF and control subjects. We identified 254 differentially expressed genes (DEGs) between IPF and controls with histologically normal lung tissue in histologically normal appearing regions of lung parenchyma. The number of DEGs remained the same (N = 254) after adjusting for cell proportions of AEC1, AEC2, fibroblasts, and ciliated cells (Fig. 3A; Table 2, Additional file 2). The majority of these DEGs (229/254; 90.1%) were upregulated in IPF subjects compared to controls with histologically normal lung tissue. Among upregulated genes in IPF are markers of senescence (*CDKN1A*) and B cell specific transcripts (*IGKC*). This was reflected in the canonical pathway analysis (Fig. 3B), with the majority of the pathways activated in IPF samples, including pathways relevant to disease processes such as epithelial adherens junction signaling [34], HIF1α signaling [35], and integrin signaling [36]. A lung-specific protein-protein interaction (PPI) network of our DEGs built in Network Analyst demonstrated enrichment for similar pathways as identified by Ingenuity among upregulated DEGs ([26]﻿; Additional file 1: Fig. S4).

**Table 2:** Summary of differential expression results (unadjusted and adjusted for cell proportions)

Comparison	# DEG unadjusted	# DEG adjusting for 4 cell types (proportions) (AEC1, AEC2, Fibroblasts, Ciliated)
IPF normal appearing lung parenchyma vs. control normal lung parenchyma	254	254
IPF normal bronchiolar vs. control normal bronchiolar	61	58
IPF transition zones of fibrosis vs. IPF normal appearing lung parenchyma	347	173
IPF dense fibrosis vs. IPF normal appearing lung parenchyma	939	198
IPF honeycomb epithelium metaplasia vs. IPF normal bronchiolar	334	1

We observed a smaller number of transcriptional differences in normal bronchiolar regions between IPF and control samples. There were 61 DEGs between IPF and controls with histologically normal lung tissue in normal bronchiolar epithelium (Fig. 3C). The number of DEGs slightly decreased (n = 58) after adjusting for cell proportions of AEC1, AEC2, fibroblasts, and ciliated cells. Ingenuity and Network Analyst enrichment analyses did not identify any enriched features, potentially due to a small number of DEGs. Among upregulated genes were DNA Damage Inducible Transcript 4 (*DDIT4*) or REDD1, a member of the same gene family as CHOP/DDIT3 which is known to be induced via HIF1α-mediated transcription [37] and is involved in negative regulation of the mammalian target of rapamycin (MTOR) signaling. Several MTOR signatures were enriched in our pathway analysis, but the direction of the gene enrichments was inconsistent. Taken together, these transcriptional changes observed in histologically non-fibrotic lung tissue and bronchiolar epithelium regions of IPF lung may represent potentially important targets for early disease detection and treatment.

### Transcriptional changes observed between histologically different regions of the IPF lung

**Fig. 4:**
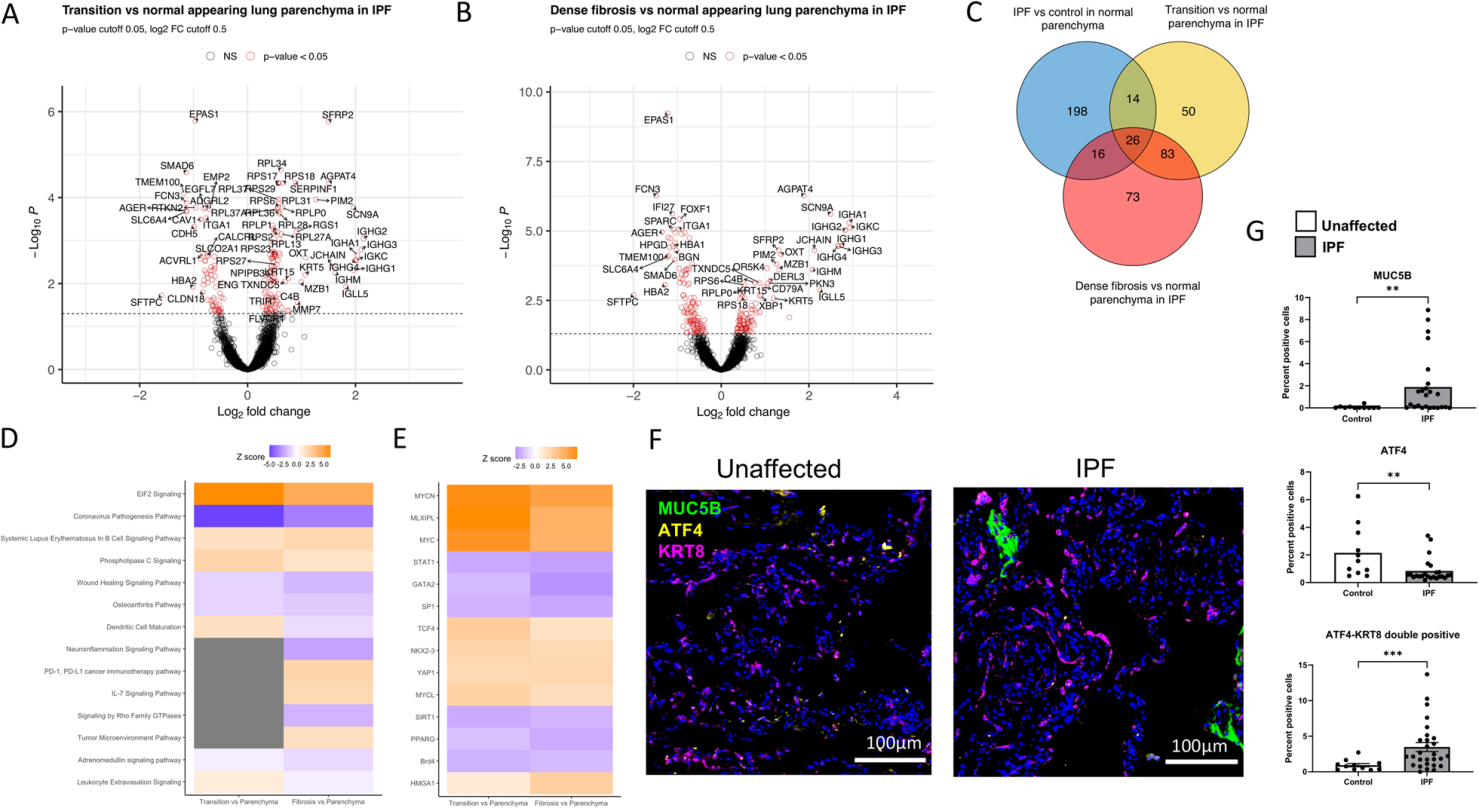
Differential expression between transition and normal appearing lung parenchyma regions (**A**) and dense fibrosis and normal appearing lung parenchyma (**B**) in IPF subjects adjusting for batch and estimated cell proportions of AEC1, AEC2, fibroblasts and ciliated cells. Venn diagram showing the overlap in DEGs between comparisons with normal lung parenchyma (**C**). Ingenuity canonical pathway (**D**) and upstream regulator analysis (**E**) of DEGs.  The activation z-score is a statistical measure based on the directional relationships between genes and their biological function. Orange indicates increased predictions over that of the null (positive z score), blue indicates decreased predictions (negative z score) and white z score of zero. Dots indicate that z score did not reach significance (1.645). Histology image showing changes in MUC5B and ATF4 (**F**). Immunofluorescence changes in MUC5B, ATF4 and ATF4 in KRT8 + cells between IPF and control in normal parenchyma regions (**G**). FOVs were chosen based on presence of features of interest (e.g. parenchyma, bronchiolar, etc.) in each section and 9 20x images centered randomly within the feature of interest were assembled into a composite. Pairwise comparisons are Mann-Whitney U test with significance set to (*) p < 0.05, (**) p < 0.01, (***) p < 0.001, (****) p < 0.0001

To determine how histologically different regions of the IPF lung diverge transcriptionally, we compared transcriptional changes in non-fibrotic IPF lung to transition areas of lung fibrosis and to areas of dense fibrosis. To understand the progression of lung tissue from non-fibrotic lung parenchyma to dense fibrosis, we individually compared non-fibrotic regions of IPF lung parenchyma to either transition regions of lung fibrosis or regions of dense fibrosis, and then explored the overlap between these comparisons. We identified 347 DEGs between transition and non-fibrotic regions; however, the number of DEGs decreased to 173 after adjusting for cell composition of AEC1, AEC2, fibroblasts, and ciliated cells (Fig. 4A; Table 2; Additional file 1: Table S3). We identified 939 DEGs between dense fibrosis regions and non-fibrotic regions, and after adjustment for cell composition, we observed 198 DEGs between regions of dense fibrosis and non-fibrotic regions of lung parenchyma (Fig. 4B; Table 2). Of the 198 DEGs between dense fibrosis and histologically normal-appearing lung, 55% (N = 109) were also differentially expressed between transition and non-fibrotic regions of lung parenchyma (Fig. 4C). Among pathways that are dysregulated in both transition and dense fibrotic areas compared to normal lung parenchyma of IPF lung are activation of the EIF2 signaling pathway and deactivation of the “wound healing signaling pathway” (Fig. 4D). Upstream regulator analysis revealed shared activation of MYC and YAP transcriptional programs with concomitant deactivation of immune-related transcriptional programs (Fig. 4E and Additional file 1: Fig. S5).

In comparing honeycomb epithelium metaplasia to normal bronchiolar epithelium in IPF lung tissue, there were 334 DEGs. However, after adjustment for cell composition, there was only a single differentially expressed gene (Additional file 1: Table S5). The largest differences in cell proportion between honeycomb epithelium metaplasia and normal bronchiolar epithelium was among ciliated cells. Examination of the 334 DEGs prior to cell adjustment confirmed strong enrichment for ciliary genes, such as GO biological process cilium assembly (GO:0060271), GO cellular component cilium (GO:0005929) and motile cilium (GO:0031514).

### Dysregulation of protein synthesis in IPF tissue

We noted enrichment of EIF2 pathway genes in normal alveolar regions from IPF patients compared to control alveoli. EIF2 is a multicomponent complex that facilitates cap-dependent mRNA translation and protein synthesis by the ribosome. Under conditions of nutrient deprivation, viral infection, and endoplasmic reticulum stress, EIF2α is phosphorylated, impairing guanine exchange and disassociation from EIF2B, inhibiting translation. The increase in EIF2 signal in IPF suggested the possibility that IPF tissue is more translationally active than control tissue. In support of this, we found increases in synthesis of the secretory cell marker, MUC5B in IPF, but not control, alveolar epithelia (Fig. 4, F and G; Additional file 1: Fig. S6).

Our previous data suggested that protein synthesis was dysregulated in mice treated with bleomycin and in individuals with IPF, and that ER stress increased in mice harboring copies of the *MUC5B* IPF risk variant [38]. However, the presence of a strong EIF2 transcriptional signature suggested against prohibitive ER stress in normal-appearing IPF tissues. To indirectly validate activation of EIF2, we stained for ATF4, which is translated by an alternative cap-independent mechanism when EIF2α is phosphorylated. Concordant with activation of EIF2 in IPF, we found that ATF4 translation was downregulated in these tissues (Fig. 4, F and G). However, we observed an increase in the number of ATF4/KRT8 double positive cells, suggesting that ATF4 is increased in these transitional alveolar stem cells [39, 40]. Taken together, these data suggest cap-dependent protein synthesis is increased in normal-appearing IPF alveoli, and that activation of the ATF4 arm of ER stress response pathways may be genotype-, or context-dependent [38].

## Discussion

Our findings indicate that the normal appearing lung in IPF is biologically abnormal and that the IPF lung includes distinct molecular patterns that are associated with the extent of lung fibrosis. In histologically normal appearing lung parenchyma and bronchiolar epithelium from the IPF lung, we have identified transcriptionally distinct patterns of gene expression, including ER stress and cell adhesion signaling, when compared to similar appearing lung tissue from controls with histologically normal lung tissue. We also show that some of the same pathways that are identified in these analyses are also differentially regulated but to a greater degree when we compared normal regions of lung parenchyma to transition zones of lung fibrosis and areas of dense lung fibrosis within the IPF lung. These findings are consistent with a recent publication that performed bulk RNA sequencing on regions of IPF lung with progressive amount of fibrosis, quantified by microCT-measured alveolar surface density (ASD) and confirmed by histology [22]. McDonough et al. identified a core set of genes increased or decreased before fibrosis was histologically evident that continued to change with advanced fibrosis. In aggregate, these findings indicate that the molecular patterns of abnormal gene expression in the IPF lung extend to normal appearing parenchyma and distal airways, and that these patterns of transcriptional activity are microscopically distinct and are associated with the extent of lung fibrosis.

The most highly activated canonical pathway that was identified in both the normal regions IPF lung and the areas of increased lung fibrosis was EIF2 signaling. EIF2α facilitates cap-dependent translation but can be phosphorylated by the unfolded protein response (UPR) activator protein PERK to promote translation of cap-independent ER stress proteins ATF4 and CHOP [41]. We observe the loss of AEC1 and AEC2 cells from normal-appearing IPF parenchyma through transition zones and dense fibrosis, with arrival of fibroblasts, myofibroblasts, and smooth muscle cells. Stimulation of collagen synthesis by fibroblasts and myofibroblasts is cap-dependent suggesting that mesenchymal cells in normal-appearing IPF tissues may give rise to the EIF2 signals we observed in our study [42]. Additional studies are needed to more fully elucidate the role of EIF2 signaling in development of lung fibrosis.

Among the activated upstream regulators are HIF1α and ATF4. Activation of pathologic ER stress pathways, including ATF4 specifically, has been identified by others as a key molecular feature of IPF [43]. In a lung fibrosis model, mice following bleomycin treatment also demonstrated activation of ER stress genes, ATF4 and ATF6, in the distal airway and honeycomb cysts [38]. Moreover, previous work has shown that HIF1α triggers ER stress and CHOP-mediated apoptosis in alveolar epithelial cells in IPF [44]. Our data show an increase of ATF4 in KRT8 + transitional alveolar stem cells.

Among upstream regulators that are activated in both transition zones and densely fibrotic areas of IPF lung are MYC and YAP transcriptional programs. Aberrant activation of YAP/TAZ has been associated with increased fibrotic remodeling in IPF in the alveolar epithelium and activated fibroblasts [45] as well as the airway epithelium [46], while the role for MYC in IPF is only emerging [47]. Among upstream regulators that are deactivated in both transition zones and densely fibrotic areas of IPF lung are immune-related transcriptional programs.

The use of the GeoMX platform to perform spatial transcriptomics on lung specimens allowed for careful selection of normal appearing lung parenchyma, as well as disease relevant regions of interest. Current single cell approaches such as droplet-based technologies capture the transcriptional information for a single cell from dissociated tissue, where the loss of spatial information can be critical to understanding the disease process. This is especially relevant in the context of a heterogenous disease of the lung, as is the case in IPF. Though the GeoMx platform does not provide single cell level information, the ability to capture normal-appearing regions within IPF, as well as transition zones which include fibroblastic foci, makes this approach advantageous to current single cell studies on dissociated lung tissue. In this study, we used two existing single cell datasets to elucidate the cellular changes between histologically distinct regions, however, direct investigation of single cell data to characterize molecular changes could be useful. Future work to utilize emerging platforms that perform spatial transcriptomics with single cell resolution will greatly improve our understanding of the early disease processes in the normal parenchyma and transition zones.

We recognize that the findings of this study could be limited to the patient demographic that was captured, predominantly males (69%), ever-smokers (66%) and NHW (94%); however, these demographics are representative of IPF [48]. Regarding the potential impact of smoking on our finding’s, smoking status was not statistically different between IPF and controls with histologically normal lung tissue (Fisher’s exact test p = 0.72).

Patients with IPF are usually diagnosed only after the fibroproliferative process has caused permanent and extensive lung parenchymal damage. Considering the irreversible nature of this disease, even approved treatments for IPF (pirfenidone [49] and nintedanib [50]) only modestly slow progression and have not been shown to alter the 3–5 year median survival after diagnosis. We have found that EIF2-dependent protein synthesis is active in normal appearing distal airways and lung parenchyma in IPF, in contrast to our previous finding that restoring normal protein synthesis in epithelia can ameliorate fibrosis [38]. This result suggests that this pathway may prove important in understanding the development of progressive lung fibrosis.

### Supplementary Information

**Additional file 1: Figure S1.** H&E stain of the 4 macroarrays of lung tissues used in this study. **Figure S2.** PCA by batch (categorical variable indicating the day tissue macroarrays were processed). **Figure S3.** Mean cell proportions estimated with SpatialDecon based on normal human lung reference. **Figure S4.** **A** Lung-specific protein-protein interactome (PPI) network of IPF vs control normal alveolar DEGs. **B** Ingenuity upstream regulator analysis of IPF vs control normal alveolar DEGs adjusted for batch and estimated cell proportions of AEC1, AEC2, fibroblasts and ciliated cells. **Figure S5.** Ingenuity upstream regulator analysis of IPF transition vs normal alveolar (**A**) and IPF dense fibrosis vs normal alveolar DEGs adjusted for batch and estimated cell proportions of AEC1, AEC2, fibroblasts and ciliated cells. **Figure S6.** Immunofluorescence changes in YAP, CDKN1A, KRT8, KRT5, SCGB3A2 and SFTPB between IPF and control in normal parenchyma regions.  All pairwise comparisons are Mann-Whitney U test with significance set to (*) p<0.05, (**) p<0.01, (***) p<0.001, (****) p<0.0001. **Table S1.**  Estimates and corresponding p-values from comparing different cell type proportions from IPF reference between regions.  Proportions were log transformed and modeled by disease region using a linear mixed model adjusting for batch and subject as a random effect.  Estimates represent the difference in log transformed proportions and p-values have been adjusted for multiple comparisons for the number of cell types using a Benjamini-Hochberg adjustment. **Table S2.**  Antiobodies and concentrations used for this study. 

** Additional file 2: Datafile S1.**  Differential expression results for main comparisons of interest (IPF normal appearing lung parenchyma vs control normal lung parenchyma, IPF normal bronchiolar vs control normal bronchiolar, IPF transition zones of fibrosis vs IPF normal appearing lung parenchyma, IPF dense fibrosis vs IPF normal appearing lung parenchyma and IPF honeycomb epithelium metaplasia vs IPF normal bronchiolar.  All gene lists are adjusted for batch and cell proportions of AECI, AECII, fibroblasts and ciliated.  ***Denotes McDonough core IPF gene.

## Data Availability

Deidentified data will be made available by request to the authors upon publication.
