# Supplementary material for: Spatially distinct molecular patterns of gene expression in idiopathic pulmonary fibrosis
Source: Respir Res. 2023 Nov 17;24:287. doi: 10.1186/s12931-023-02572-6 (PMC10655274; doi:10.1186/s12931-023-02572-6)
Supplement: Supplementary file 1 — Additional file 1: Figure S1. H&E stain of the 4 macroarrays of lung tissues used in this study. Figure S2. PCA by batch (categorical variable indicating the day tissue macroarrays were processed). Figure S3. Mean cell proportions estimated with SpatialDecon based on normal human lung reference. Figure S4. A Lung-specific protein-protein interactome (PPI) network of IPF vs control normal alveolar DEGs. B Ingenuity upstream regulator analysis of IPF vs control normal alveolar DEGs adjusted for batch and estimated cell proportions of AEC1, AEC2, fibroblasts and ciliated cells. Figure S5. Ingenuity upstream regulator analysis of IPF transition vs normal alveolar (A) and IPF dense fibrosis vs normal alveolar DEGs adjusted for batch and estimated cell proportions of AEC1, AEC2, fibroblasts and ciliated cells. Figure S6. Immunofluorescence changes in YAP, CDKN1A, KRT8, KRT5, SCGB3A2 and SFTPB between IPF and control in normal parenchyma regions. All pairwise comparisons are Mann-Whitney U test with significance set to (*) p<0.05, (**) p<0.01, (***) p<0.001, (****) p<0.0001. Table S1. Estimates and corresponding p-values from comparing different cell type proportions from IPF reference between regions. Proportions were log transformed and modeled by disease region using a linear mixed model adjusting for batch and subject as a random effect. Estimates represent the difference in log transformed proportions and p-values have been adjusted for multiple comparisons for the number of cell types using a Benjamini-Hochberg adjustment. Table S2. Antiobodies and concentrations used for this study. [file 12931_2023_2572_MOESM1_ESM.docx]

**Spatial resolution of transcriptional heterogeneity in idiopathic pulmonary fibrosis**

**Additional Figures**

Figure S1. H&E stain of the 4 macroarrays of lung tissues used in this study.

_
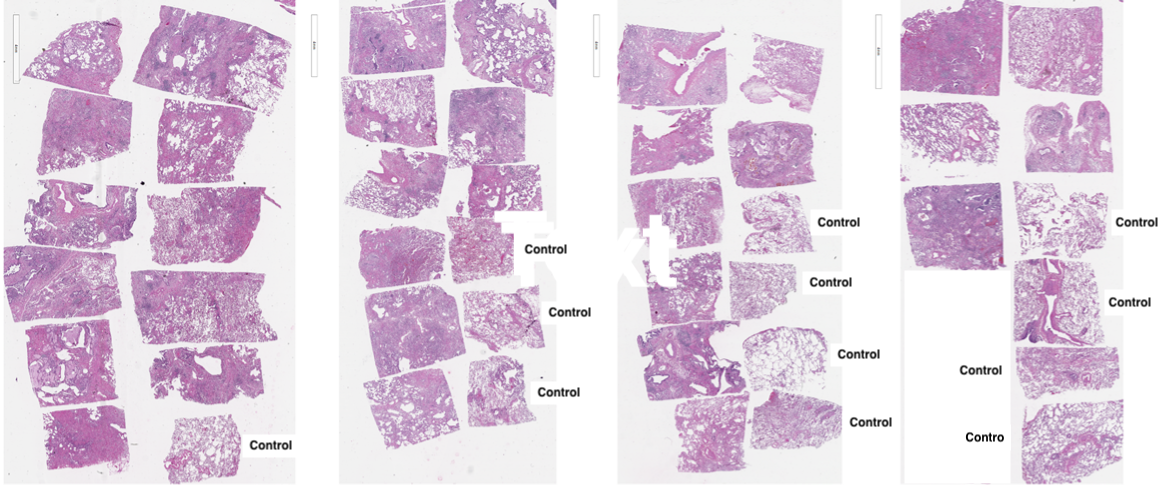
_

Figure S2. PCA by batch (categorical variable indicating the day tissue macroarrays were processed).

Figure S3. Mean cell proportions estimated with SpatialDecon based on normal human lung reference.

Figure S4. (A) Lung-specific protein-protein interactome (PPI) network of IPF vs control normal alveolar DEGs. (B) Ingenuity upstream regulator analysis of IPF vs control normal alveolar DEGs adjusted for batch and estimated cell proportions of AEC1, AEC2, fibroblasts and ciliated cells.

A


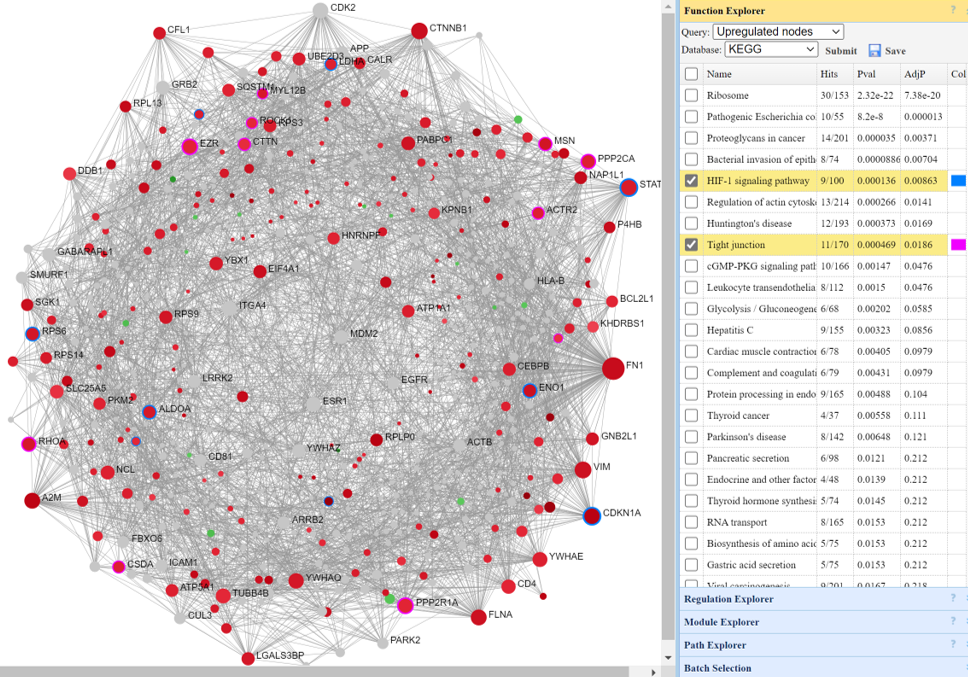
A

B


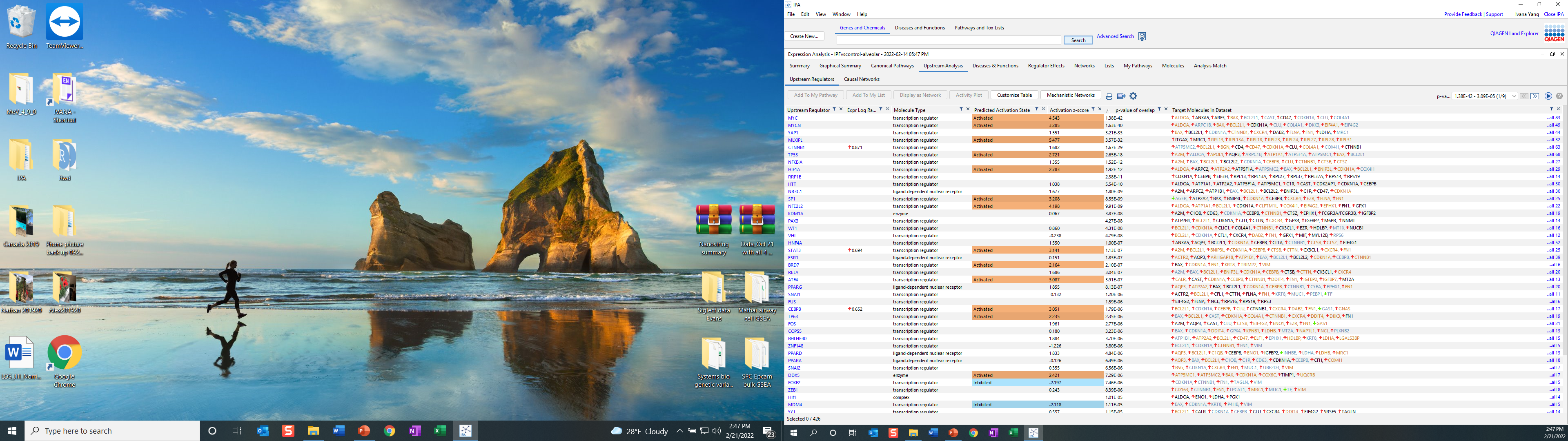


Figure S5. Ingenuity upstream regulator analysis of IPF transition vs normal alveolar (A) and IPF dense fibrosis vs normal alveolar DEGs adjusted for batch and estimated cell proportions of AEC1, AEC2, fibroblasts and ciliated cells.

A


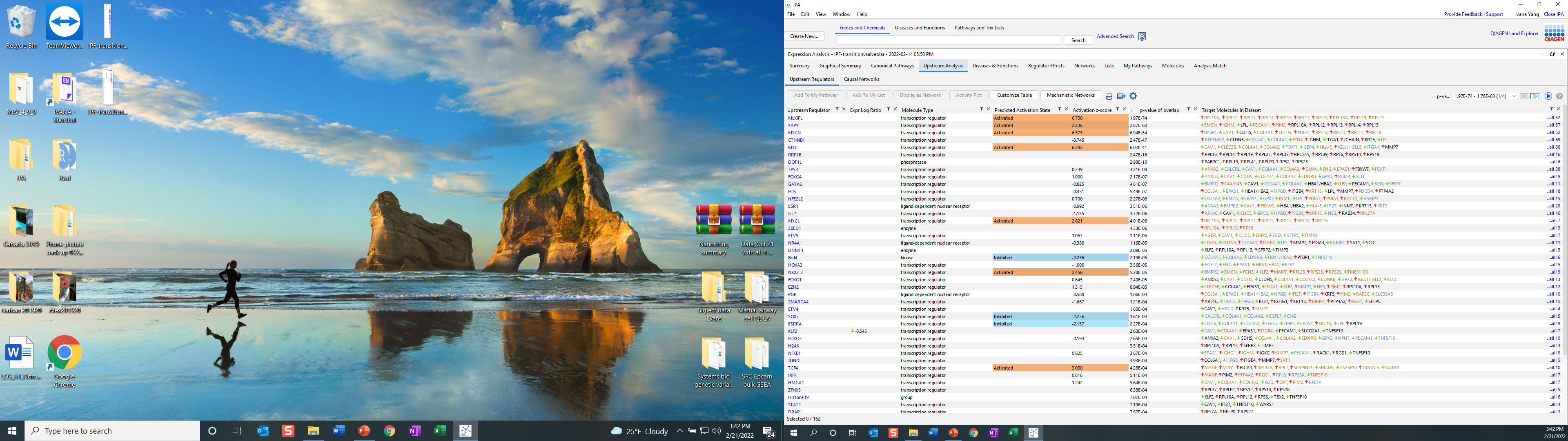


B


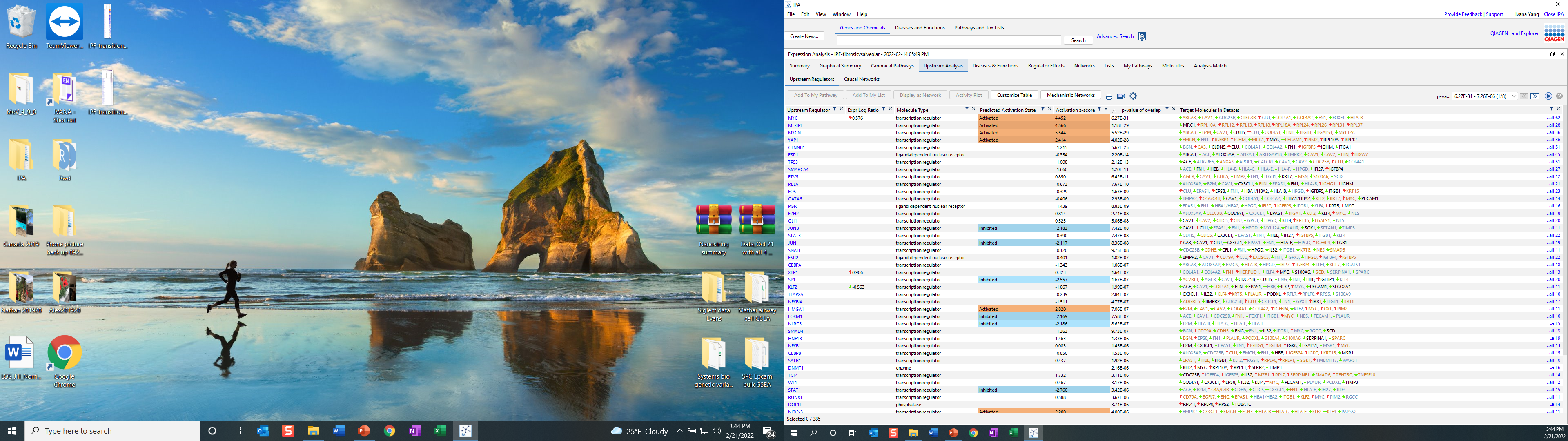


Figure S6. Immunofluorescence changes in YAP, CDKN1A, KRT8, KRT5, SCGB3A2 and SFTPB between IPF and control in normal parenchyma regions. All pairwise comparisons are Mann-Whitney U test with significance set to (*) p<0.05, (**) p<0.01, (***) p<0.001, (****) p<0.0001.

Table S1. Estimates and corresponding p-values from comparing different cell type proportions from IPF reference between regions. Proportions were log transformed and modeled by disease region using a linear mixed model adjusting for batch and subject as a random effect. Estimates represent the difference in log transformed proportions and p-values have been adjusted for multiple comparisons for the number of cell types using a Benjamini-Hochberg adjustment.

|  | IPF parenchyma vs controls parenchyma | | IPF bronchial vs controls bronchial | | IPF transition vs IPF parenchyma | | IPF dense fibrosis vs IPF parenchyma | | IPF honeycomb vs IPF bronchial | |
| --- | --- | --- | --- | --- | --- | --- | --- | --- | --- | --- |
| Celltype | Est. | p-value | Est. | p-value | Est. | p-value | Est. | p-value | Est. | p-value |
| Aberrant_Basaloid | 0.31 | 0.537 | 0.97 | 0.22 | 0.91 | 8e-04 | -0.09 | 0.72 | 0.76 | 0.062 |
| ATI | -0.19 | 0.639 | -0.32 | 0.48 | -0.99 | 9e-05 | -2.03 | 2e-15 | 0.58 | 0.123 |
| ATII | -0.39 | 0.537 | -1.60 | 0.12 | -0.91 | 0.005 | -1.32 | 3e-05 | 0.58 | 0.274 |
| B_Plasma | 0.71 | 0.170 | 0.07 | 0.90 | 0.64 | 4e-04 | 1.34 | 1e-13 | 0.52 | 0.062 |
| Basal | 0.38 | 0.501 | 1.21 | 0.17 | 0.13 | 0.619 | -0.32 | 0.29 | 0.49 | 0.281 |
| Ciliated | -1.57 | 0.007 | -0.50 | 0.48 | 0.21 | 0.587 | -0.15 | 0.67 | -1.90 | 3e-04 |
| Fibroblast | -0.52 | 0.496 | -0.60 | 0.45 | 0.63 | 0.041 | 1.94 | 4e-10 | -0.10 | 0.853 |
| Myofibroblast | 0.45 | 0.126 | 0.03 | 0.90 | 0.84 | 2e-08 | 0.55 | 1e-04 | 0.83 | 2e-04 |
| Goblet | 0.66 | 0.408 | 0.87 | 0.31 | 0.28 | 0.434 | -0.24 | 0.51 | -0.96 | 0.062 |
| Lymphatic | -0.64 | 0.211 | -0.52 | 0.45 | 0.79 | 0.006 | 2.09 | 3e-12 | -0.25 | 0.621 |
| Macrophage | 0.48 | 0.486 | 0.98 | 0.24 | 0.34 | 0.320 | 0.20 | 0.56 | 1.39 | 0.003 |
| Macrophage_Alveolar | 0.05 | 0.900 | 0.47 | 0.39 | -0.28 | 0.224 | -0.55 | 0.01 | 1.03 | 0.002 |
| Mast | 0.28 | 0.418 | -0.58 | 0.22 | 0.52 | 0.001 | 0.92 | 5e-09 | 0.56 | 0.021 |
| NK | -1.03 | 0.124 | -0.82 | 0.31 | -0.15 | 0.619 | -0.61 | 0.06 | -0.26 | 0.621 |
| Pericyte | 1.80 | 7e-05 | 0.43 | 0.48 | -1.96 | 2e-10 | -1.63 | 2e-08 | 0.27 | 0.621 |
| SMC | -0.12 | 0.660 | -0.41 | 0.31 | 0.29 | 0.042 | 0.86 | 3e-09 | -0.11 | 0.621 |
| T_Cytotoxic | 0.03 | 0.937 | -1.05 | 0.23 | 0.94 | 0.005 | 0.67 | 0.04 | -0.01 | 0.973 |
| VE_Capillary_A | -0.15 | 0.805 | -0.80 | 0.31 | -1.70 | 1e-07 | -1.95 | 8e-10 | 0.44 | 0.396 |
| VE_Capillary_B | -0.41 | 0.501 | -0.51 | 0.45 | -0.89 | 0.002 | -0.73 | 0.01 | 0.55 | 0.258 |

Table S2. Antiobodies and concentrations used for this study.

| **Target** | **Mfg** | **Clone/ref** | **Origin species/target species** | **FFPE** |
| --- | --- | --- | --- | --- |
| ATF4 | Proteintech | 60035-1AP | Mouse anti-human mAb | 1:500 |
| SFTPC | Abcam | ab90716 | Rabbit anti-human pAb | 1:200 |
| SFTPB | Invitrogen | MAI-204 | Mouse anti-human mAb | 1:200 |
| AGER | R&D | AF1179 | Goat anti-human mAb | 1:200 |
| KRT5 | Covance/Biolegend | 905901 | Chicken anti-human/mouse pAb | 1:400 |
| KRT8 | Millipore | MABT329/TROMA1 | Rat anti-human mAb | 1:200 |
| SCGB3A2 | R&D | AF3545 | Goat anti-human pAb | 1:200 |
| MUC5B | Novus | 50390 | Mouse anti-human mAb | 1:200 |
| MUC5B | Sigma | HPA008246-100uL | Rabbit anti-human pAb | 1:200 |
| CDKN1A | Proteintech | 10355 | Rabbit anti-human pAb | 1:100 |
| YAP | Millipore | MABS2029 | Rat anti-human mAb | 1:200 |

Datafile S1. Differential expression results for main comparisons of interest (IPF normal appearing lung parenchyma vs control normal lung parenchyma, IPF normal bronchiolar vs control normal bronchiolar, IPF transition zones of fibrosis vs IPF normal appearing lung parenchyma, IPF dense fibrosis vs IPF normal appearing lung parenchyma and IPF honeycomb epithelium metaplasia vs IPF normal bronchiolar. All gene lists are adjusted for batch and cell proportions of AECI, AECII, fibroblasts and ciliated. ***Denotes McDonough core IPF gene
